# Primary headache disorders in the adult general population of Pakistan – a cross sectional nationwide prevalence survey

**DOI:** 10.1186/s10194-017-0734-1

**Published:** 2017-02-23

**Authors:** A. A. Herekar, A. Ahmad, U. L. Uqaili, B. Ahmed, J. Effendi, S. Z. Alvi, M. A. Shahab, U. Javed, A. D. Herekar, Rafiq Khanani, T. J. Steiner

**Affiliations:** 1 Headache Research Foundation of Pakistan, Karachi, Pakistan; 2 Department of Surgery, University of Texas at San Antonio, San Antonio, TX USA; 3 Dow University of Health Sciences, Karachi, Pakistan; 4 Department of Neurology, Center for Movement Disorders and Neurorestoration, University of Florida College of Medicine, Gainesville, FL USA; 5 Department of Medicine, University of Nevada, Las Vegas, USA; 6 Aga Khan Medical College, Aga Khan Medical University Hospital, Karachi, Pakistan; 7 School of International and Public Affairs, Columbia University, New York, NY USA; 8 Department of Neurology, Baqai Medical University, Karachi, Pakistan; 9 Department of Pathology and Microbiology Department at Dow University of Health Sciences, Karachi, Pakistan; 10 Department of Neuroscience, Norwegian University of Science and Technology (NTNU), Edvard Griegs Gate, NO-7491 Trondheim, Norway; 11 Division of Brain Sciences, Imperial College London, London, UK

**Keywords:** Headache disorders, Migraine, Tension-type headache, Medication-overuse headache, Epidemiology, Prevalence, Population-based survey, Eastern Mediterranean Region, Pakistan, Global campaign against headache

## Abstract

**Background:**

The large geographical gaps in our knowledge of the prevalence and burden of headache disorders include almost all of Eastern Mediterranean Region (EMR). We report a nationwide population-based study in Pakistan, an EMR country with the sixth largest population in the world, conducted as a project within the Global Campaign against Headache.

**Methods:**

We surveyed six locations from the four provinces of Pakistan: Punjab, Sindh, Khyber Pakhtunkhwa and Baluchistan. We randomly selected and visited rural and urban households in each. One adult member (18–65 years) of each household, also randomly selected, was interviewed by a trained non-medical interviewer from the same location using a previously-validated structured questionnaire translated into Urdu, the national language. We estimated 1-year prevalences of the headache disorders of public-health importance and examined their associations with demographic variables using multivariate analysis.

**Results:**

There were 4223 participants (mean age 34.4 ± 11.0 years; male 1957 [46.3%], female 2266 [53.7%]; urban 1443 [34.2%], rural 2780 [65.8%]). Participation proportion was 89.5%. Headache in the previous year was reported by 3233 (76.6% [95% CI: 75.3–77.8%]). The age- and gender-adjusted 1-year prevalence of migraine was 22.5% [21.2–23.8%] (male 18.0% [16.8–19.2%], female 26.9% [25.6–28.2%]), of tension-type headache (TTH) 44.6% [43.1–46.1%] (male 51.2% [49.7–52.7%], female 37.9% [36.4–39.4%]), of probable medication-overuse headache 0.7% [0.5–1.0%] (male 0.7% [0.5–1.0%], female 0.8% [0.5–1.1%]) and of other headache on ≥15 days/month 7.4% [6.6–8.2%] (male 4.4% [3.8–5.0%], female 10.4% [9.5–11.3%]). Migraine was more prevalent in females by a factor of 3:2 although this association barely survived (*P* = 0.039) after correcting for other factors. TTH was more prevalent in males by about 4:3 (*P* = 0.026). All headache and migraine were age-related, peaking in the age group 40–49 years; TTH peaked a decade earlier. Higher education (*P* = 0.004) and income (*P* = 0.001) were negatively associated with prevalence of migraine.

**Conclusion:**

With three quarters of its population affected, headache disorders must be on the public-health agenda of Pakistan. Worldwide, these disorders are the third leading cause of disability; information from specific enquiry into the burden attributable to headache disorders in this country is needed to inform health policy and priority-setting, and will be reported soon.

## Background

Headache disorders affect almost half the world’s population, according to a survey of the published literature conducted nine years ago [1]. Tension-type headache (TTH) and migraine are the major contributors in terms of prevalence, being the second and third most common disorders in the world [2]. In 2013, migraine was recognised as the sixth-highest cause of disability in the world [3]. Also important in public-health terms, because of the burdens they impose at individual level, are the group of disorders characterized by headache on ≥15 days/month; these include medication-overuse headache (MOH), not itself a primary headache disorder but, in almost all cases, a *sequela* due to mismanagement of either migraine or TTH [4]. MOH is the 18^th^-highest cause of disability in the world [3].

Large geographical gaps in our knowledge of the prevalence and burden of headache disorders have been evident from the various surveys [1, 2, 5, 6]. In a continuing endeavour to fill these, *Lifting The Burden* (LTB), a United Kingdom-based non-governmental organisation conducting the Global Campaign against Headache [7] in official relations with the World Health Organization [8], has been supporting population-based studies in many parts of the world: among others, in Russia in Eastern Europe [9], in China in the Western Pacific Region [10] and in India [11] and Nepal [12] in South East Asia. Over 2.5 billion people live in these countries, where knowledge was virtually absent. In the Eastern Mediterranean Region is another large geographical knowledge gap; the countries include Pakistan, with the sixth largest population in the world [13] and characterised by economic and political instability and, in parts, by geographical inaccessibility.

We report here the prevalence results of a nationwide cross-sectional population-based survey in Pakistan. It was conducted as part of the series of similar studies within the Global Campaign against Headache, and following the standardized methodology developed by LTB for such studies [14]. It is the first to be published from the Region. Its two purposes were to contribute to knowledge of the global burden of headache [3] and to provide evidence for national health policy in Pakistan.

## Methods

The detailed methodology has been published previously [15]. Here the methods are summarized.

### Ethics

The Ethics Review Board of the Dow University of Health Sciences approved the study protocol. All participants were informed about the nature and purpose of the survey and gave their consent to taking part. Data protection legislation was complied with.

### Survey

We conducted the survey in six locations purposively selected from the four provinces of Pakistan to represent the national population: Lahore and Multan (Punjab), Karachi and Sukkur (Sindh), Abbottabad (Khyber Pakhtunkhwa) and Gwadar (Baluchistan). Rural and urban households randomly selected in each location were visited unannounced by a team of 12 trained non-medical interviewers recruited from, and therefore familiar with, the same locations. One randomly-selected adult member (18–65 years) of each household was interviewed using LTB’s structured HARDSHIP questionnaire translated into Urdu, the national language. This questionnaire, used in similar studies conducted in other countries [16], included demographic enquiry, screening and diagnostic questions for headache. Additionally, weight and height were measured, and body mass index (BMI) calculated.

### Diagnosis

Diagnoses were not made by the interviewers, but subsequently by diagnostic algorithm [16], applied to the most bothersome headache if a participant reported more than one type of headache. The diagnostic questions had been validated earlier in a Pakistani population [15]. Cases were removed for individual review of medication use when headache was reported on ≥15 days/month, and diagnosed either as probable MOH (pMOH) or other headache on ≥15 days/month. All remaining cases (episodic headache) were classified by applying modified ICHD-II criteria in hierarchical sequence: first definite migraine, then definite TTH, then probable migraine and finally probable TTH. Cases falling into none of these categories were unclassified. During subsequent analysis, definite and probable migraine were combined, as were definite and probable TTH, for generating prevalence estimates for migraine and TTH. The correctness of this approach has been argued [14].

### Data management

Data from completed questionnaires were entered into SPSS version 16.0. We applied full double data-entry by two operators working independently, subsequently eliminating errors by reference to the original forms.

### Statistics

We planned a total sample size of 4149. In calculating this we assumed a headache prevalence of 50% and applied a confidence level of 99% and confidence interval (CI) of 2%.

Data analysis was conducted using Stata/SE 12.0 and SPSS v 23. Continuous variables were summarized as means and standard deviations (SDs), and categorical variables as numbers and percentages.

We categorised age as 18–29, 30–39, 40–49, 50–59 or 60–65 years; marital status as “single”, “married” or “divorced, widowed or separated”; habitation as “urban” or “rural”; educational level as “none”, “some schooling” (up to high school) or “college” (including university); income level as “poor” or “not poor” (“poor” meaning below the poverty line [30^th^ population income percentile, or income per quarter of < PKR 9,000 or USD 90] [17]); BMI according to the WHO classification [18] as “underweight” (<18.5), “normal” (18.5–24.9), “overweight” (25.0–29.9), “obese” (30–39.9) or “morbidly obese” (>40).

We used chi-squared to compare distributions between categorical variables. We calculated headache prevalences as percentages with 95% CIs. We performed bivariate analyses, calculating odds ratios (ORs) with 95% CIs, to look for associations between headache types (migraine, TTH or all headache on ≥15 days/month) and demographic variables. In subsequent multivariate logistic regression analyses, we calculated Exp(B) with 95% CIs taking prevalence of each headache type as the dependent variable and gender, age, marital status, habitation, education level, income, province and BMI as factors. Model-fitting statistics indicated a good fit (chi-squared = 501.98, df = 60, *P* < 0.001). In the principal (prevalence) and bivariate association analyses we set statistical significance at *P* < 0.05; in the multivariate analysis, because of multiple comparisons we set it at *P* < 0.02.

### Quality assurance

We applied preventative, detective and corrective quality assurance procedures as described elsewhere [15]. During the data verification process, irregularities in data collection were identified in one location, Multan, and rectified, which necessitated discarding the original (fraudulent) data and repeating the survey in this location (a full account of this has been published elsewhere [19]). The results reported here include the data from the second survey in Multan.

## Results

The survey was completed by 4223 participants (1957 [46.3%] male, 2266 [53.7%] female) aged 18–65 years (mean 34.4 ± 11.0), of whom 1443 (34.2%) lived in urban and 2780 (65.8%) in rural areas. The participation proportion was 89.5% overall, with regional variation between 69% and >99%. The sample was generally well matched to the population of all Pakistan for gender, age and urban/rural habitation according to figures from Pakistan’s last census (1998) [20], albeit with males and the over-50 age groups slightly under-represented. Most participants (72.1%) were married, 26.0% were unmarried and only 1.9% were divorced, separated or widowed; the latter two groups were also slightly under-represented. During bivariate analysis, prevalences were adjusted for age and gender by weighting according to population data [20] since these were likely to be influencing factors.

Observed prevalences of all headache and of the specific headache types, overall and by gender, are shown in Table 1. Headache of any sort (“all headache”) in the past year was reported by 3233 participants, an observed 1-year prevalence of 76.6%. There was no difference between males and females. TTH was by far the most prevalent headache disorder (44.7%), but migraine was also very common, reported by over one fifth (22.9%) of participants. Headache on ≥15 days/month was reported by almost one in 12 participants (8.1%), of whom a small minority (0.7%) were diagnosed as pMOH. Only 37 cases (0.9%) were unclassifiable, similar numbers in each gender (Table 1). Migraine was more prevalent in females in a ratio of 3:2 (*P* < 0.001), pMOH and other headache on ≥15 days/month about two-fold (the latter significantly [*P* < 0.001] but the former not). TTH, on the other hand, was more prevalent in males by a factor of about 4:3 (*P* < 0.001) (Table 1).

**Table 1:** Observed and age- and gender-adjusted 1-year prevalences of all headache and headache types

Headache type	Observed overall (*N* = 4223) (%) [95% CI]	Age-adjusted (%) [95% CI]		Age- and gender-adjusted (*N* = 4223) (%) [95% CI]
		Males (*n* = 1957)	Females (*n* = 2266)	
All headache	76.6 [75.3–77.9]	75.4 [74.1–76.7]	76.8 [75.5–78.1]	76.1 [74.8–77.4]
Migraine	22.9 [21.6–24.2]	18.0 [16.8–19.2]	26.9 [25.6–28.2]	22.5 [21.2–23.8]
Tension-type headache	44.7 [43.2–46.2]	51.2 [49.7–52.7]	37.9 [36.4–39.4]	44.6 [43.1–46.1]
Probable medication-overuse headache	0.7 [0.5–1.0]	0.7 [0.5–1.0]	0.9 [0.6–1.2]	0.8 [0.5–1.1]
Other headache on ≥15 days/month	7.4 [6.6–7.9]	4.4 [3.8–5.0]	10.4 [9.5–11.3]	7.4 [6.6–8.2]
Unclassifiable	0.9 [0.6–1.2]	1.2 [0.9–1.5]	0.8 [0.5–1.1]	1.0 [0.7–1.3]

*CI* confidence interval. Adjusted for age and gender in SPSS v23 by weighting in accordance with the formula Weight = proportion in population/proportion in sample, taking population data from [20]

**Fig. 1:**
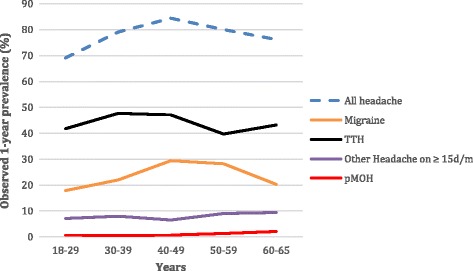
Observed 1-year prevalences of headache types by age (TTH: tension-type headache; d/m: days/month; pMOH: probable medication-overuse headache)

Relationships with age are shown in Fig. 1 and Table 2. The curves for all headache and migraine show prevalence increasing to a peak during the age range 40–49 years, then declining; prevalence of TTH peaked 10 years earlier but remained high in the 5^th^ decade. The observed prevalence of pMOH increased steadily with age, although numbers were too small to show a significant relationship.

**Table 2:** Observed prevalences of migraine and tension-type headache, by age and gender

Age group (years)	One-year prevalence % (n)			
	Male		Female	
	Migraine	TTH	Migraine	TTH
18–29	15.3% (110)	47.4% (342)	20.1% (164)	36.8% (300)
30–39	17.8% (74)	53.0% (220)	24.6% (162)	44.5% (293)
40–49	20.6% (90)	59.2% (258)	38.3% (164)	34.8% (149)
50–59	20.7% (60)	47.9% (139)	36.4% (96)	30.7% (81)
60–65	19.4% (24)	47.6% (59)	22.1% (15)	35.3% (24)

*TTH* tension-type headache

Table 3 shows the bivariate analyses. Married participants had more overall headache (OR = 1.8) and migraine (OR = 1.5) than those who were single. Participants who were divorced, widowed or separated also had more headache (OR = 1.9), migraine (OR = 1.8) and headache on ≥15 days/month (OR = 2.5), but less TTH (OR = 0.7) than single participants. No significant associations emerged between marital status and pMOH.

**Table 3:** Bivariate analyses† to demonstrate potential associations between headache types and demographic factors, habitation and body mass index

Variable (*N* = 4223)	Odds ratios (95% confidence intervals)				
	All headache (*n* = 3233)	Migraine (*n* = 967)	Tension-type headache (*n* = 1887)	Probable MOH (*n* = 30)	Other headache on ≥15 d/m (*n* = 312)
Age					
18–29 years (*n* = 1537)	Reference	Reference	Reference	Reference	Reference
30–39 years (*n* = 1074)	1.8 (1.5–2.1)*	1.3 (1.1–1.6)*	1.3 (1.1–1.5)*	0.7 (0.2–2.1)	1.1 (0.9–1.5)
40–49 years (*n* = 865)	2.5 (2.1–3.1)*	1.8 (1.4–2.3)*	1.2 (1.1–1.5)*	1.1 (0.4–3.0)	0.9 (0.7–1.3)
50–59 years (*n* = 554)	1.9 (1.5–2.4)*	1.8 (1.4–2.3)*	0.9 (0.8–1.1)	2.0 (0.7–5.2)	1.3 (0.9–1.8)
60–65 years (*n* = 193)	1.5 (1.1–2.1)*	1.2 (0.8–1.7)	1.1 (0.8–1.4)	3.3 (1.0–10.5)*	1.4 (0.8–2.3)
Marital status (*N* = 4182; missing 41)					
Single (*n* = 1309)	Reference	Reference	Reference	Reference	Reference
Married (*n* = 2637)	1.8 (1.5–2.1)*	1.5 (1.3–1.8)*	1.1 (1.0–1.3)	1.8 (0.7–4.5)	1.1 (0.8–1.4)
Divorced, widowed or separated (*n* = 236)	1.9 (1.4–2.7)*	1.8 (1.3–2.5)*	0.7 (0.5–0.9)*	-	2.5 (1.6–3.7)*
Habitation (*N* = 4223)					
Rural (*n* = 2780)	Reference	Reference	Reference	Reference	Reference
Urban (*n* = 1443)	1.0 (0.9–1.2)	1.2 (1.0–1.4)	0.7 (0.6–0.8)*	1.9 (0.9–4.2)	1.8 (1.4–2.3)*
Educational level (*N* = 3263; missing 960)					
None (*n* = 600)	Reference	Reference	Reference	Reference	Reference
Some schooling (*n* = 2454)	1.2 (1.0–1.4)	0.5 (0.4–0.7)*	2.2 (1.8–2.6)*	1.2 (0.4–3.6)	0.6 (0.4–0.8)*
College (*n* = 209)	0.6 (0.4–0.8)*	0.5 (0.3–0.7)*	1.5 (1.1–2.1)*	-	0.4 (0.2–0.8)*
Income level (*N* = 3606; missing 617)					
Poor (*n* = 1127)	Reference	Reference	Reference	Reference	Reference
Not poor (*n* = 2479)	0.7 (0.6–0.9)*	0.8 (0.7–0.9)*	1.3 (1.1–1.5)*	0.8 (0.3–1.7)	0.4 (0.3–0.5)*
Body mass index (*N* = 3889; missing 334)					
Normal (*n* = 2152)	Reference	Reference	Reference	Reference	Reference
Underweight (*n* = 155)	0.6 (0.4–1.0)*	1.7 (1.1–2.5)*	0.4 (0.3–0.7)*	1.8 (0.4–8.2)	1.2 (0.6–2.1)
Overweight (*n* = 1156)	1.0 (0.8–1.2)	1.2 (1.0–1.4)	0.9 (0.7–1.0)	0.9 (0.4–2.2)	1.0 (0.7–1.3)
Obese (*n* = 417)	0.6 (0.5–0.8)*	1.6 (1.3–2.1)*	0.4 (0.3–0.5)*	0.3 (0.04–2.1)	1.5 (1.0–2.2)*
Morbidly obese (*n* = 9)	0.2 (0.15–0.25)*	1.6 (1.3–2.1)*	0.4 (0.3–0.5)*	1.1 (0.3–4.0)	0.7 (0.4–1.2)

*MOH* medication-overuse headache, *d/m* days/month. †Adjusted in SPSS v23 for age and/or gender (as appropriate) by weighting in accordance with the formula Weight = proportion in population/proportion in sample, taking population data from [20]. *P < 0.05

Urban dwelling was weakly positively associated with migraine (OR = 1.2) and more strongly with other headache on ≥15 days/month (OR = 1.8) but negatively with TTH (OR = 0.7) (Table 3).

Increasing educational level was negatively associated with migraine and other headache on ≥15 days/month, and positively with TTH (Table 3). Accordingly, being not poor was negatively associated with migraine (OR = 0.8) and strongly so with other headache on ≥15 days/month (OR = 0.4), and with all headache (OR = 0.7), but positively with TTH (OR = 1.3) (Table 3).

BMI was calculated for 3889 participants (for 334, either height or weight was not recorded). Only a little over half of participants (2,152; 55.5%) were of normal weight: over 40% were overweight (1156; 29.8%) or obese (417; 10.7%) and nine participants were morbidly obese. The extremes (underweight and obese) were negatively associated with all headache and TTH but positively with migraine; obesity was also positively associated with headache on ≥15 days/month (Table 3).

In multivariate analysis (Table 4), the positive association between female gender and migraine barely survived (*P* > 0.02), but the association with headache on ≥ 15 days/month remained strongly significant, as did the negative association with TTH. Age 40–49 years remained positively associated with migraine, and an association emerged with headache on >15 days/month, while ages 30–49 were positively associated with TTH. Migraine was confirmed as more prevalent among married participants, but otherwise there were no associations with marital status. No associations with area of habitation survived. Some schooling was positively associated with TTH whereas college education continued to be strongly negatively associated with migraine. No other associations with educational level survived. Being poor remained strongly associated with both migraine and headache on ≥15 days/month, but the negative association with TTH did not survive. There were regional variations: relative to the Punjab, migraine was much less common in Khyber Pakhtunkhwa and TTH was less common in all other regions, but especially in Khyber Pakhtunkhwa; on the other hand, headache on ≥15 days/month was more common in Sindh and Baluchistan. Being underweight and being obese each remained negatively associated with TTH, but no other associations with BMI survived.

**Table 4:** Multivariate analysis of associations between headache types and demographic and environmental factors and body mass index

Variable	Exp(B) (95% confidence intervals)		
	Migraine (*n* = 967)	Tension-type headache (*n* = 1887)	Headache on ≥15 d/m (*n* = 342)
Gender (*N* = 4223) compared to male (*n* = 1957)			
Female (*n* = 2266)	1.3 (1.01–1.7) *P* = 0.039	0.8 (0.7–0.97) *P* = 0.026	2.0 (1.4–2.9) ***P*** **< 0.001**
Age (*N* = 4223) compared to 18–29 years (*n* = 1507)			
30–39 years (*n* = 1197)	0.9 (0.6–1.3) *P* = 0.522	1.5 (1.1–2.0) ***P*** **= 0.012**	1.2 (0.7–2.0) *P* = 0.536
40–49 years (*n* = 1101)	2.0 (1.3–2.9) ***P*** **= 0.001**	2.4 (1.7–3.4) ***P*** **< 0.001**	2.0 (1.2–3.5) ***P*** **= 0.015**
50–59 years (*n* = 330)	1.1 (0.6–2.1) *P* = 0.720	1.4 (0.8–2.3) *P* = 0.201	2.0 (0.9–4.4) *P* = 0.076
60–65 years (*n* = 88)	2.1 (0.7–6.7) *P* = 0.201	2.8 (1.1–7.1) *P* = 0.034	1.8 (0.3–9.4) *P* = 0.515
Marital status (*N* = 4182; missing 41) compared to single (*n* = 1089)			
Married (*n* = 3015)	1.8 (1.2–2.5) ***P*** **= 0.002**	1.3 (0.9–1.7) *P* = 0.115	1.2 (0.7–2.0) *P* = 0.490
Divorced, widowed or separated (*n* = 78)	0.5 (0.1–1.8) *P* = 0.277	0.8 (0.3–2.0) *P* = 0.683	1.4 (0.5–4.3) *P* = 0.568
Habitation (*N* = 4223) compared to rural (*n* = 2780)			
Urban (*n* = 1443)	1.3 (1.0–1.7) *P* = 0.053	1.1 (0.9–1.3) *P* = 0.554	1.0 (0.7–1.5) *P* = 0.895
Educational level (N = 3277; missing 946) compared to none (*n* = 166)			
Some schooling (*n* = 2691)	0.8 (0.5–1.2) *p* = 0.263	1.8 (1.2–2.9) ***p*** **= 0.011**	1.3 (0.7–2.4) *p* = 0.503
College (*n* = 420)	0.4 (0.2–0.8) ***p*** **= 0.004**	1.0 (0.6–1.7) *p* = 0.959	0.9 (0.4–2.0) *p* = 0.721
Income level (*N* = 3606; missing 617) compared to poor (*n* = 1127)			
Not poor (*n* = 2479)	0.5 (0.4–0.7) ***p*** **< 0.001**	0.8 (0.6–1.01) *p* = 0.061	0.3 (0.2–0.5) ***p*** **< 0.001**
Province (*N* = 4223) compared to Punjab (*n* = 2431)			
Sindh (*n* = 1017)	1.0 (0.8–1.4) *P* = 0.781	0.7 (0.5–0.9) ***P*** **= 0.002**	2.2 (1.5–3.4) ***P*** **< 0.001**
Baluchistan (*n* = 201)	0.5 (0.2–0.9) *P* = 0.022	0.5 (0.3–0.9) ***P*** **= 0.011**	2.7 (1.2–5.9) ***P*** **= 0.013**
Khyber Pakhtunkhwa (*n* = 574)	0.2 (0.1–0.3) ***P*** **< 0.001**	0.4 (0.3–0.6) ***P*** **< 0.001**	0.5 (0.3–1.1) *P* = 0.077
Body mass index (*N* = 3889; missing 334) compared to normal (*n* = 2152)			
Underweight (*n* = 155)	0.8 (0.4–1.4) *P* = 0.412	0.5 (0.3–0.8) ***P*** **= 0.005**	1.4 (0.7–2.9) *P* = 0.386
Overweight (*n* = 1156)	1.2 (0.9–1.6) *P* = 0.193	1.0 (0.8–1.3) *P* = 1.00	1.3 (0.8–1.9) *P* = 0.298
Obese or morbidly obese (*n* = 426)	0.9 (0.6–1.4) *P* = 0.573	0.4 (0.3–0.6) ***P*** **< 0.001**	1.4 (0.8–2.6) *P* = 0.249

Significant associations (*P* < 0.02) are emboldened. *d/m* days/month

## Discussion

This first-ever nationwide population-based study of the prevalence of headache disorders in Pakistan (indeed, in any country in the Eastern Mediterranean Region) has revealed much headache in the country. The overall 1-year prevalence of 76.6% is substantially higher than the reported global average of 46% [1]. At 22.9% and 44.7%, the prevalences of migraine and TTH each greatly exceed their respective global estimates of 14.7% and 20.7% from the Global Burden of Disease Survey 2010 [2]. Headache on ≥15 days/month is highly prevalent (8.1%), although only 0.7% of the adult population have pMOH.

With 4223 participants, a participation proportion of 89.5%, a representative sample and a well-performing diagnostic instrument [15], these estimates from Pakistan have low margins of uncertainty. The key methodological limitation was that we could not reach most slum-dwellers, who are inevitably excluded from household surveys while making up an estimated 45.5% of the urban population according to some statistics [21]. These are people below the poverty line, in whom, if the association with poverty holds (see below), headache will be common.

This study was one of a series being conducted by LTB in pursuit of the Global Campaign against Headache. While several of these are not yet completed or fully analyzed, the careful enquiry incorporated into the methodology of these studies is finding that headache prevalences have in the past been significantly underestimated. For example, very similar findings have emerged from India, a country which shares Pakistan’s genetic, environmental and cultural composition: the 1-year prevalence in Karnataka State of all headache was 63.9%, of migraine 25.2% (with a 4:3 female preponderance) and of TTH 35.1% [22]. Also in Nepal, a similar nationwide study found 1-year prevalences of all headache of 84.9%, of migraine 34.1% and of TTH 41.5% [12], and in Russia, another similar study found prevalences of 62.9% for all headache, 20.8% for migraine and 30.8% for TTH [9]. It would therefore be incorrect to suggest, although they are very common in Pakistan, that headache in general and migraine in particular are excessively prevalent in this country. The reality is that these are very common disorders worldwide, to a degree that is only now being recognised. Only in the Far East does evidence persist of lower prevalence: for example, 23.8% for all primary headache and 9.3% for migraine from a similar nationwide study supported by LTB in China [10]. Whether the reasons for this are genetic, environmental or cultural is yet to be ascertained.

The prevalence in Pakistan of all causes of headache on ≥15 days/month (8.1%) is approaching three times the estimated global average of 3% [1], but still not outside the range found by similar studies. It is similar in Nepal (7.4%) [12] and Georgia (7.6%) [23], and appreciably higher in Russia (10.4%) [9], but lower in India (3.0%) [22]. In Russia, but apparently not Georgia, much of this is associated with medication overuse, and therefore reported as pMOH, with prevalences of 7.2% and 0.9% respectively. In India, the prevalence of pMOH is 1.2% [22] and in Nepal it is 2.1% [12] against Pakistan’s 0.7%. Pakistan therefore mirrors Georgia – also a lower-middle-income country – in this respect. This level of highly-frequent headache in Pakistan must be a cause of concern, for it signals much public ill health. A cross-sectional survey cannot establish the causes: these require investigation in clinical settings, although, as in Georgia, medication overuse does not appear to be the major factor. Studies elsewhere suggest multiple factors [24], including high consumption of caffeine (which is present in black tea, typically consumed many times a day in Pakistan) and various health-related issues such as asthma, hypothyroidism and hypertension. Pakistan’s unrecognized public ill health and disability warrant enquiry into these factors.

There were some associations of interest. As everywhere [1], observed prevalence of migraine was higher in females than in males, reflecting a female predisposition understood to be hormonally determined [25]. However, the ratio was only 1.5:1 (26.9% versus 18.0%) and multivariate analysis found female gender, with Exp(B) = 1.3, to be only barely significant (*P* = 0.039). It may be noted, as a plausible explanation, that the culture of Pakistan expects women’s interviews to be conducted in their husbands’ presence while, at the same time, discouraging women from expressing pain in front of their husbands (neurologists in Pakistan are familiar with anecdotal accounts of husbands seeking divorce from their wives on account of their “constant complaints” of headache). In other words, migraine may have been somewhat under-reported by women. Speculatively, the same may have occurred, to a greater degree, with TTH, a disorder perhaps more easily hidden than migraine: in contrast to most studies elsewhere, we found in Pakistan a significantly higher prevalence of TTH in males (51.2%) than in females (37.9%), although this difference, with Exp(B) = 0.8, was also barely significant (*P* = 0.026) after correction for other factors. Comment is still needed on why the prevalence of TTH in males (51.2%) is unusually high – more than in India, and even Nepal, where prevalence was again higher in males (44.6%) than females (38.7%) [12]. This finding occurred consistently in every city surveyed except Sukkur (n = 81). The male:female working ratio is very high in Pakistan [26]; the burden of supporting the entire household falls almost solely on the male. Whether or not this is a relevant factor may emerge in future studies in other mostly patriarchal countries: preliminary results from Saudi Arabia (unpublished) show a similar trend.

Age had differential impacts on headache types and in the genders. For migraine, the trends were slightly different in males and females, with a more significant effect seen in females (Table 2). Migraine in females increased from adolescence (18–29 years) to middle age (30–49), declining slightly in the perimenopausal years (50–59) and sharply decreasing in the postmenopausal age group of 60–65. These trends can be attributed to the well-documented influence on migraine of oestrogen levels [25]. For TTH, prevalence peaked later in males (40–49) than in females (30–39).

The associations with educational level were complex and not easily explained, except to the extent that they matched those of income level (these two factors themselves being strongly correlated, although each retained some independent associations with the probabilities of migraine and TTH in multivariate analysis [Table 4]). Poverty by itself was positively associated with migraine and headache on ≥15 days/month and negatively with TTH, although the last observation did not hold true after multivariate analysis. In general, therefore, it seems migraine is associated with lower socioeconomic status (SES), being significantly less prevalent among the college-educated and not poor. Educational and income levels affect lifestyle, diet, stress and general hardship levels and, of course, general health and access to health care, so that the social causation hypothesis is speculatively invoked to explain this association [27]: lower SES brings stress and other disease mediators that increase the incidence or duration of illness. The association between poverty and headache has been noted elsewhere [9, 23, 28].

The differences between provinces are noteworthy. Punjab and Sindh have more migraine than Khyber Pakhtunkhwa and, probably, Baluchistan, whereas Punjab has more TTH than all other provinces. Yet headache on ≥15 days/month is more common in Sindh and Baluchistan than in Punjab or, especially, Khyber Pakhtunkhwa. These differences are not easily explained. Each province is largely occupied by a different ethnic group: Punjabi, Sindhi, Baloch and Pashtun, although there are many minority groups. Punjab is an agricultural province with warmer weather; it has higher average SES, which appears contradictory to the trend for migraine but in line with that for headache on ≥15 days/month. Khyber Pakhtunkhwa, which has least headache of all types, has more rural dwellings and a colder climate, and much of it is at high altitudes. In Nepal, increasing altitude, at least between sea level and 2,500 m, is correlated with increasing migraine prevalence [12]. Khyber Pakhtunkhwa is also beset by armed conflict and terrorism, which might be expected to increase rather than reduce headache levels. These differences warrant further investigation.

Associations with BMI were also complex, those of migraine and TTH again appearing to be in opposite directions. Such associations have been studied extensively [29–31]. Possible mechanisms to link migraine and headache on ≥15 days/month with obesity have been described [30] and will not be commented on here. From a public-health perspective, it is of interest if obesity is a risk factor for these disorders since it is potentially modifiable (unlike age, gender and poverty). However, the positive association of obesity with migraine did not survive multivariate analysis, whereas the *negative* association with TTH remained strong (Exp(B) 0.4; *P* < 0.001). Previous studies [32] have been unable to link TTH with BMI. Overall, not much can be made of these findings with respect to BMI, although it might be observed that obesity is quickly becoming a public-health problem in Pakistan [33].

There are limitations to our study. First, all weightings relied on data that are almost twenty years old given that Pakistan’s last census was in 1998 [20]. Second, as noted earlier, we could not reach almost 45% of the urban population who were slum-dwelling [21], which potentially introduced a bias. Third, because of the security situation in Pakistan during the conduct of this study, we were unable to access Quetta, the largest city in Baluchistan province, and areas north of Abbotabad in the Khyber Pakhtunkhwa province. The same concern limited us from going into the deep rural population. Whether these restrictions led to appreciable bias is unknown.

What is the importance of these findings to Pakistan? It is clear that headache disorders in this country are extremely prevalent in absolute terms, and high also relative to global means. These facts were not previously known. But Pakistan has many and considerable public-health problems [32], and prevalence alone does not establish a case for priority. In this regard it can be noted that migraine is the sixth highest cause of disability in the world [3] and Pakistan has much more migraine than the world on average. Burden data will be reported in a future publication.

## Conclusion

With over three quarters of its population affected, headache disorders must be on the public-health agenda of Pakistan. Worldwide, these disorders are the third leading cause of disability [6]; information from specific enquiry into the burden attributable to headache disorders in this country is needed to inform health policy and priority-setting, and will be reported soon.
